# A dual role of EphB1/ephrin-B3 reverse signaling on migrating striatal and cortical neurons originating in the preoptic area: should I stay or go away?

**DOI:** 10.3389/fncel.2014.00185

**Published:** 2014-07-18

**Authors:** Judith Rudolph, Katrin Gerstmann, Geraldine Zimmer, André Steinecke, Annika Döding, Jürgen Bolz

**Affiliations:** Institut für Allgemeine Zoologie und Tierphysiologie, Universität JenaJena, Germany

**Keywords:** interneuron guidance, stop signal, neuronal migration, cortical interneurons, striatum, ephrins, Src, wiring molecules

## Abstract

During embryonic development the preoptic area (POA) gives rise to two populations of neurons which are generated at the same time, cortical interneurons and striatal cells. POA-derived cortical interneurons take a superficial path and avoid the developing striatum (Str) when they migrate to their target region. We found that EphB1, which is expressed in the striatal anlage, prevents cortical interneurons from entering the Str via ephrin-B3 reverse signaling. In contrast, for striatal neurons which also express ephrin-B3, EphB1 acts as a stop signal. This dual role of EphB1 is due to differences in ephrin-B3 reverse signaling cascades. For striatal neurons, binding of EphB1 to ephrin-B3 reduces endogenously high levels of pSrc and pFAK, which then causes the cells to stop migration. In contrast, in cortical interneurons EphB1-ephrin-B3 reverse signaling leads to phosphorylation of Src and focal adhesion kinase (FAK) which then mediates repulsion. Consistent with these *in vitro* findings, in an ephrin-B3 knockout mouse line, we discovered misrouted cortical interneurons in the Str and an over-migration of striatal neurons in their target region. Thus, EphB1/ephrin-B3 reverse signaling has a different impact on two sets of neurons which are generated at the same time and place: it can act as a repulsive cue for migrating neurons or it can terminate neuronal migration, a novel role of the Eph/ephrin system.

## Introduction

Inhibitory interneurons modulate the homeostasis between excitation and inhibition, precisely gating information and consequently shaping neuronal circuits (Klausberger and Somogyi, 2008). Given that interneurons only account for 20–25% of all cortical neurons, they are essential for modulating mature neocortical brain function (Druga, 2009). An imbalance in this proportion causes instability of neuronal circuits which can lead to neurological and mental diseases (Marín, 2012). Whereas excitatory projection neurons are generated in the proliferative zones of the dorsal telencephalon and migrate radially along the glial fibers to form the laminated neocortex (Rakic and Komuro, 1995; Malatesta et al., 2000; Nadarajah et al., 2001), inhibitory cortical interneurons originate from the medial and caudal ganglionic eminences (MGE and CGE, respectively) as well as from the preoptic area (POA) of the basal telencephalon. Then they accomplish a long range tangential migration along a deep and a superficial migratory stream (SMS) through the basal telencephalon into the developing cortex (de Carlos et al., 1996; Tamamaki et al., 1997; Marín and Rubenstein, 2001; Nakajima, 2007; Marín et al., 2010; Guo and Anton, 2014). This migration is precisely regulated by several brain wiring molecules (Bolz and Castellani, 1997), many of which have been originally described as axonal guidance cues.

One important group of these molecules are the ephrins and the Eph receptor tyrosine kinases. A distinctive feature of this protein family is that its signaling can be induced in forward, i.e., based on typical ephrin ligand and Eph receptor interactions, but also in reverse, when Eph receptors activate ephrin ligands (Davy and Soriano, 2005). Many members of the Eph/ephrin system show a spatially and temporally distinct expression in the developing brain (Peuckert et al., 2008). Initially, ephrins were discovered as regulators for the formation of topographic projections (Cheng et al., 1995; Drescher et al., 1995), but it is now well established that they play many additional roles in the development of the nervous system (e.g., Lisabeth et al., 2013; Klein and Kania, 2014 for recent reviews). In the context of interneuron migration, ephrin-A5 acts as a repellent cue that forces the exit of newborn interneurons from the ventricular zone (VZ) of the MGE (Zimmer et al., 2008). In addition, repulsive ephrinA3/EphA4 interactions prevent MGE derived cortical interneurons expressing EphA4 from entering the striatum (Str), a non-target region for these neurons, where ephrin-A3 is expressed (Rudolph et al., 2010). The Eph/ephrin system is also involved in the selection of specific migratory routes of interneurons to the cortex (Zimmer et al., 2011). Finally, a very recent study provided evidence that Eph/ephrin signaling can also act as a motogenic signal for migrating cortical interneurons that promotes the migration of cortical interneurons (Steinecke et al., 2014).

In the present study we examined the effects of EphB1/ephrin-B3 reverse signaling on neurons generated in the POA. This was motivated by the complementary expression patterns of ephrin-B3, which is expressed in the POA and in the migratory stream of cortical interneurons, and EphB1, which is expressed in non-target regions of these neurons, including the Str. We found that EphB1 has a repulsive effect on migrating cortical interneurons mediated by ephrin-B3, which prevents these neurons from entering the Str. Surprisingly, a population of striatal neurons which are generated in the POA with cortical interneurons simultaneously bear ephrin-B3. However, for these cells EphB1 acts as a stop signal, indicating to them that their journey is complete. To decipher how the same ligand/receptor combination can lead to such diverse responses in these two sets of neurons, we examined the EphB1/ephrin-B3 signaling cascades in cortical and striatal neurons. Previous studies provided evidence that Src, a member of the Src-family-kinases (SFKs), is often associated with ephrin-B ligands and mediates reverse signaling (Palmer et al., 2002; Zimmer et al., 2011). In addition, FAK and Src often act in a complex in which FAK becomes autophosphorylated at Tyr397 and then binds and activates Src (Mitra et al., 2005; Wu et al., 2008). We found that the levels of phosphorylated Src and FAK (pSrc and pFAK) are regulated in striatal and cortical neurons differentially. In striatal cells, binding of EphB1 to ephrin-B3 leads to a reduction of the endogenously high pSrc and pFAK levels which causes the cells to terminate their migration. Reduction of pSrc or pFAK levels in these cells mimicked the effects of EphB1 and led to a migration arrest. In contrast, in cortical interneurons binding of EphB1 to ephrin-B3 leads to phosphorylation of Src and FAK which then mediates repulsion. Finally, we performed *in vivo* studies in an ephrin-B3 knockout mouse line and found abnormalities in the migration of cortical and striatal neurons that are consistent with the data from our *in vitro* assays.

## Materials and methods

### Mouse strains

Wild-type (WT) mice maintained in a C57BL/6 background were used for expression analysis, dissociated single-cell experiments, the outgrowth assay and in organotypic slice cultures. Homozygous ephrin-B3 knock-out mice (Kullander et al., 2001), maintained at the C57BL/6 background (received from Dr. R. Klein, Max Planck Institute for Neurobiology, Martinsried, Germany), were used to obtain ephrinB3 knock-out embryos. Genotyping was confirmed by genomic PCR. For staging of mouse embryos, the day of insemination was considered as embryonic day 1 (E1). Mice were bred and maintained under standard conditions and were kept with access to food and water *ad libitum* on a 12-h light/dark cycle. All animal procedures were performed in agreement with the institutional regulations of the University of Jena (Jena, Germany).

### *In situ* hybridization

Digoxigenin (DIG)-labeled RNA-probes for EphB1 (609–1480 of mouse Ephb1, GenBank accession number NM_173447) and ephrin-B3 (137–955 of mouse Ephrinb3, GenBank accession number NM_007911) were used for *in situ* hybridization. Heads of E14 WT embryos were freshly frozen in liquid nitrogen and 18 μm coronal cryostat sections were thaw-mounted on SuperFrost Plus slides (Thermo Fisher Scientific). *In situ* hybridization was performed as described previously (Rudolph et al., 2010). In brief, sections were dried 2–3 h at 56°C, fixed for 10 min in 4% paraformaldehyde (PFA) in Diethyl pyrocarbonate (DEPC)-treated 1 M PBS (pH 7.4) and permeabilized in 0.2 M HCl for 10 min, and acetylated in 0.1 M triethanolamine with 5 mM acetanhydrid for 15 min. Sections were hybridized overnight at 72°C using a probe concentration of 3 ng/μl. After blocking with 2% blocking reagent (Roche, Germany) for 2–3 h DIG-labeled riboprobe hybrids were detected using an anti-DIG Fab fragment conjugated with alkaline phosphatase (1:750; Roche, Germany). For color reaction a mixture of 5-Bromo-4-chloro-3-indolyl phosphate (Roche, Germany) and Nitro blue tetrazolium chloride (Roche, Germany) in reaction buffer was used 4–6 h for EphB1 and overnight for ephrin-B3 at room temperature.

### Immunochemistry

Immunohistochemistry was performed on 18 μm coronal cryosections of E14 and E16 embryonic brains that were immersion fixed with 4% PFA in PBS for 4 h at room temperature. Fixed brains were then cryoprotected overnight with 15% and 30% sucrose at 4°C before freezing in liquid nitrogen for cryosectioning. Slices were postfixed with 4% PFA in PBS for 2 min, incubated in epitope retrieval solution (Leica) at 70°C for 2 min and treated with the blocking reagent (4% BSA, 10% normal goat serum, in PBS with 0.2% Triton X-100) for 2 h before incubating with the primary antibody overnight at room temperature. For staining against calbindin retrieval was not necessary. Secondary antibodies were applied for 2 h at room temperature. Nuclei were stained with DAPI (100 ng/ml in PBS; Sigma-Aldrich) for 15 min. For immunocytochemistry in dissociated cells or explants, blocking occurred for 1 h in 4% BSA, 10% normal goat serum in PBS with 0.2% Triton X-100. Incubation with the primary antibody diluted in blocking reagent was performed overnight at 4°C for dissociated cells and at room temperature for explants, respectively. Probes were then incubated for 1.5 h with the secondary antibodies and stained with DAPI (100 ng/ml in PBS; Sigma-Aldrich) for 20 min at room temperature. For double or triple immunochemistry, the primary or secondary antibodies were supplied as a mixture. Primary antibodies used: rabbit anti-calbindin (Swant; 1:1000), mouse anti-Islet-1 (developmental studies hybridoma bank; 1:200), rabbit anti-pSrc (pY418) (Biosource; 1:250), rabbit anti-pFAK (pY397) (invitrogen; 1:500), rabbit anti-pTyr (PY350) (Santa Cruz; 1:500), rabbit anti-Lhx6 (Santa Cruz; 1:100). Secondary antibodies were as follows: Cy3 goat anti-rabbit IgG (1:2000), Cy2 goat anti-rabbit IgG (1:300 for calbindin; 1:100 for pSrc), Cy3-goat anti-mouse IgG (1: 2000) (all from Jackson ImmunoResearch Laboratories), goat anti-mouse IgG Alexa633 (1:500, Invitrogen).

### Preparation of dissociated neurons

Time pregnant mice were deeply anesthetized using peritoneal injection of 10% chloralhydrate. The E14 mouse embryos were removed from the uterus and the brains were cut coronally into 225 μm slices using a tissue chopper and transferred into Gey’s balanced salt solution (GBSS) supplemented with 0.65% glucose. Only brain slices including the POA, MGE and lateral ganglionic eminence (LGE) were chosen. After dissection of the POA domain, the ventricular zone/subventricular zone (VZ/SVZ) and intermediate zone (IMZ) of the MGE were prepared. The tissue was collected in ice-cold Hank’s balanced salt solution (HBSS; Invitrogen, Germany) supplemented with 0.65% glucose. After incubation with 2.5% trypsine in HBSS for 17 min at 37°C, the tissue was dissociated into single cells by trituration and filtered through nylon gauze to remove cell aggregates. Neurons were seeded at a density of 300 cells/mm^2^ and incubated in Dulbeccós Modified Eagle Medium (DMEM; Invitrogen, Germany) supplemented with 10% fetal bovine serum (FBS), 10,000 U/ml penicillin, 10,000 μg/ml streptomycin, 0.065% D-glucose and 0.4 mM L-glutamine at 37°C and 5% CO_2_ in a humid atmosphere for 2 days. Cells were fixed in 4% PFA in PBS for 15 min at room temperature.

### Binding assay

Primary neurons seeded at a density of 300 cells/mm^2^ were grown for 24 h, then 5 μg/ml recombinant EphB1-Fc (R and D Systems) preclustered with 20 μg/ml goat anti-human IgG Alexa488 (Invitrogen) or goat anti-human IgG Alexa546 was applied for 30 min at 37°C and 5% CO_2_ in fresh cell culture medium. Then cells were washed briefly in warm PBS and fixed with 4% PFA in PBS.

### Preparation of organotypic slice cultures

Brains of E14 embryos of WT mice were prepared in Krebs’ buffer (126 mM NaCl, 2.5 mM KCl, 1.2 mM NaH_2_PO_4_, 1.2 mM MgCl_2_, 2.1 mM CaCl_2_, 10 mM D-glucose, 12.5 mM NaHCO_3_), embedded in 4% low melt agarose (Roth) in Krebs’ buffer at 37°C and subsequently cut coronally into 300 μm slices using a vibratome at 4°C. Slices were transferred into ice-cold postholding buffer (Krebs’ buffer with 10 mM HEPES, 10,000 U/ml penicillin, 10,000 μg/ml streptomycin and 50 μg/ml gentamicin), then slices including the POA, MGE and LGE were placed on Nucleopore polycarbonate culture membranes and incubated in serum-free medium composed of 60% DMEM/F12 (Sigma), 30% HBSS, 10,000 U/ml penicillin, 10,000 μg/ml streptomycin and 0.65% D-glucose for 1 h at 37°C and 5% CO_2._ Then tungsten beads coated with CellTracker C2925 were placed into the IMZ of the slices using a capillary. After 2 h at 37°C and 5% CO_2_ medium was changed to medium containing 10% FBS. For blocking endogenous ephrin-B ligands, 5 μg/ml recombinant EphB1-Fc or, as a control, 5 μg/ml Fc protein, were added to the medium. The organotypic slice cultures were incubated for 1 days *in vitro* (DIV) at 37°C and 5% CO_2_ in a humid atmosphere, and then fixed with 4% PFA in PBS for 2 h at room temperature.

### Stripe assay

Stripe assays were performed according to Vielmetter et al. (1990) using silicone matrices, obtained from the Max-Planck Institute for Developmental Biology (Tübingen, Germany), for stripe formation. Glass coverslips were placed on the silicone matrix and 25 μl of a 10 μg/ml EphB1-Fc solution preclustered with 30 μg/ml anti-human IgG-Alexa488 or anti-human Alexa546 in PBS were injected into the matrix channels. After incubation at 37°C for 30 min, the coverslips were washed with PBS and coated with 19.5 μg/ml laminin (Sigma-Aldrich) and 5 μg/ml poly-L-lysine (Invitrogen) for 30 min, to obtain alternating stripes of labeled EphB1-Fc and unlabeled control protein (laminin-poly-L-lysine). To assemble higher concentrated stripes we used 50 μg/ml EphB1-Fc preclustered with 80 μg/ml anti-human IgG-Alexa488. As a control experiment alternating stripes of 3 μg/ml Fc (human IgG F(c) fragment; Rockland Immunochemicals) clustered with 30 μg/ml anti-human IgG-Alexa488 in PBS, and laminin-poly-L-lysine were used. Dissociated neurons were added at a density of 750 cells/mm^2^ and grown for 2 DIV at 37°C and 5% CO_2_ in cell culture medium. To reduce endogenous Src levels, 5 μM of the SFK-inhibitor proteinphosphatase 2 (PP2; Calbiochem) or 5 μM of the control peptide PP3 (Calbiochem) were added to the medium. To activate Src, 5 μM Src family activator (Santa Cruz) were used. To reduce endogenous FAK levels, 3 μM FAK-inhibitor 14 (Santa Cruz) were added to the medium.

### siRNA transfection

For siRNA transfection of MGE-neurons reverse lipofection with Lipofectamine RNAiMAX (Invitrogen) was used according to the manufacturer’s protocol. MGE-derived neurons growing on alternating stripes of EphB1-Fc and control protein were transfected with 10 nM mouse ephrin-B3 siRNA, containing a pool of 3 target-specific 20–25 nt siRNAs to knock down gene expression (Santa Cruz), in combination with 10 nM Alexa555-labeled RNA dublex (BLOCK-iT Alexa Fluor red fluorescent oligo; Invitrogen) to enable the visualization of the transfected interneurons. Transfection occurred for 5 h in antibiotics-free cell culture medium at 37°C and 5% CO_2_ in a humid atmosphere. Then the medium was substituted by culture medium containing 10,000 U/ml penicillin and 10,000 μg/ml streptomycin. Transfected neurons were incubated for 2 DIV at 37°C and 5% CO_2_.

### Verification of ephrin-B3 silencing after siRNA transfection

For validation of the ephrin-B3 silencing by siRNA, NIH3T3 fibroblasts transfected with a retroviral vector, pLIG, containing the human ephrin-B3 full length cDNA and a Geneticin G418 resistance were grown in DMEM/F12 containing 10% FBS, 1% penicillin/streptomycin and 0.4% Geneticin G418. Confluent petridishes were transfected with 200 pmol ephrin-B3 siRNA combined with 200 pmol Alexa555 RNA dublex using Lipofectamine2000 (Invitrogen) according to the manufacturer’s protocol for 4 h. After 24 h pictures were taken to determine the transfection efficiency. The RNA was isolated with Trizol (Roth) according to the manufacturer’s instructions. For cDNA synthesis the RevertAid MinusM-MuLV Reverse Transcriptase (Fermentas) was used (42°C for 60 min). PCR against ephrin-B3 (forward, GGGATATGGAAGCTTTGAGAC; reverse, GGTATCACCACCCACAACCAGC) and actin (forward, AGAGGGAAATCGTGCG; reverse, CAATAGTGATGACCTGGCCGT) was performed. To quantify the expression, ephrin-B3 bands were normalized against actin.

### Verification of inhibition of pFAK after treatment with FAK-inhibitor 14

To proof the reduction of the pFAK level by FAK-inhibitor 14, cells dissected from whole E14 brains were cultured 2 DIV at 37°C and 5% CO_2_ in cell culture medium supplemented with 1 mM, 3 mM or 7.5 mM FAK-inhibitor 14. The medium was renewed after 1 DIV. Then cells were lysed in STEN buffer (150 mM NaCl, 50 mM Tris, 2 mM EDTA, and 0.2% NP-40) including broad spectrum-protease inhibitor (Sigma) and phosphatase inhibitor (PhosStop, Roche). Lysates were separated on NuPAGE 4–12% Bis-Tris Gels (life technologies) following the manufacturer’s instructions and transferred to nitrocellulose membranes. Membranes were blocked in TBS-T buffer (300 mM NaCl, 10 mM Tris, pH 7.6, and 0.1% Tween20) containing 5% milk-powder for 30 min and then incubated with the primary antibody over night at room temperature. Antibodies used were mouse anti-actin (Santa Cruz, 1:1000); rabbit anti-pFAK (pY397) (Invitrogen; 1:500); rabbit anti-FAK (Millipore; 1:1000). After washing in TBS-T, membranes were incubated for 1 h at room temperature with biotin-conjugated secondary antibodies (goat anti-mouse, Sigma; 1:400 and goat anti-rabbit, Vector; 1:400). Membranes were washed again in TBS-T and the signal was detected using DAB detection kit (Vector laboratories) following the manufacturer’s instructions. The total FAK amount was normalized to actin and pFAK bands were then normalized to the normalized FAK.

### Outgrowth assay

The POA and IMZ domain of E15 WT brains was dissected from 225 μm slices, collected in ice-cold MC medium (0.4 g methyl cellulose, 1% penicillin / streptomycin, 1% L-glutamine, 0.1% glucose in DMEM) and cut in 200 × 200 μm pieces using a tissue chopper. The MGE explants were precultured at 37°C and 5% CO_2_ for 1 h, embedded in chicken plasma (Sigma) that was cross-linked with thrombine (Sigma) and cultured in MC medium. After 20 h *in vitro* the medium was replaced with MC medium containing either 5 μg/ml recombinant EphB1-Fc or, as a control, 5 μg/ml Fc protein, or the Src inhibitor PP2 (5 μM) with PP3 (5 μM) as the according control. After 2 DIV cultures were fixed with 4% PFA for 45 min and an immunostaining against Isl-1 followed.

### Microscopy

Pictures of* in situ* hybridizations and dissociated single cells were taken using a Zeiss Axiovert S100 inverted microscope combined with a digital camera (Spot; Diagnostic Instruments). For *in situ* hybridizations a 5× objective (Zeiss; PlanNeofluar; NA 0.15) was used. For quantification and documentation of the immunohistochemistry in cryosections a 10× objective (Zeiss PlanNeofluar, NA 0.6) and 1.6 optovar or a 20× phase contrast objective (Zeiss; PlanNeofluar, NA 0.5) was used. For the stripe assay, pictures were taken using a 20× phase contrast objective and 1.6 optovar in combination with fluorescence excitation to visualize the stripes. Pictures of migrating CellTracker-labelled cells in the slice assay were taken with a Zeiss Laser Scanning Microscope (LSM) 510 and ZEN 2009 software (Zeiss) using a 10× objective (Zeiss, NA 0.6) and an argon laser with 488 nm wavelength. A band-pass of 500–550 nm was applied as a filter for emission. For the analysis of fluorescence intensities of pSrc stained cells as well as for single cells in the EphB1-binding assay pictures were taken with the LSM510 and ZEN 2009 software using a 40× immersion water objective (Zeiss; C-Apochromat; NA 1.2), an argon laser with the settings as described above and a helium-neon laser (wavelength 543 nm) in combination with an emission long-pass filter set (560 nm). DAPI-stained nuclei were visualized using a tuneable two-photon titanium-sapphire laser (Mai-Tai; Spectra Physics) with a wavelength of 780 nm and a 435–485 nm band-pass filter set. For the four color stained cells using Cy2, Alexa546 and Alexa633 secondary antibodies and DAPI, pictures were taken with the LSM510 and ZEN 2009 software as well as the 40× immersion water objective. Cy2 was detected using an argon laser with an absorption wavelength of 488 nm and an emission band-pass of 500–550 nm. Alexa546 was detected with a helium-neon laser (wavelength 543 nm) in combination with an emission band-pass filter set (565–615 nm), and Alexa633 using a helium-neon laser (wavelength 633 nm) with a long-pass filter (650 nm). For all fluorophores the main beam splitter UV/488/543/633 nm was used. For DAPI-stained nuclei the Mai-Tai laser was used as described before. The extraction of a single optical plane along the x- and y-axis was performed with the ZEN 2009 software (ortho-view).

### Quantitative analysis of *in vitro* assays

Individual images of migrating cells in the slice assay were automatically composed with the *Stiching*-function of the ZEN 2009 software. The migration pattern in the LGE was analyzed by determining the cell numbers of the migrated CellTracker labelled cells across a sector ranging from the VZ to the ventral border of the LGE using *ImageJ* (W. S. Rasband, National Institutes of Health, Bethesda, MD). This sector was divided vertically into 10 equal segments, with segments 1–4 representing the proliferating zones (VZ and SVZ), segments 5–7 the striatal anlage and segments 8–10 the piriform cortex as visualized in Figure 2D. The relative cell number per segment was calculated in relation to the total number of migrated cells for each slice. One way-*ANOVA* variance analysis was used for statistical comparison (*R* software). The number of analyzed brain sections is indicated as “n”.

**Figure 1:**
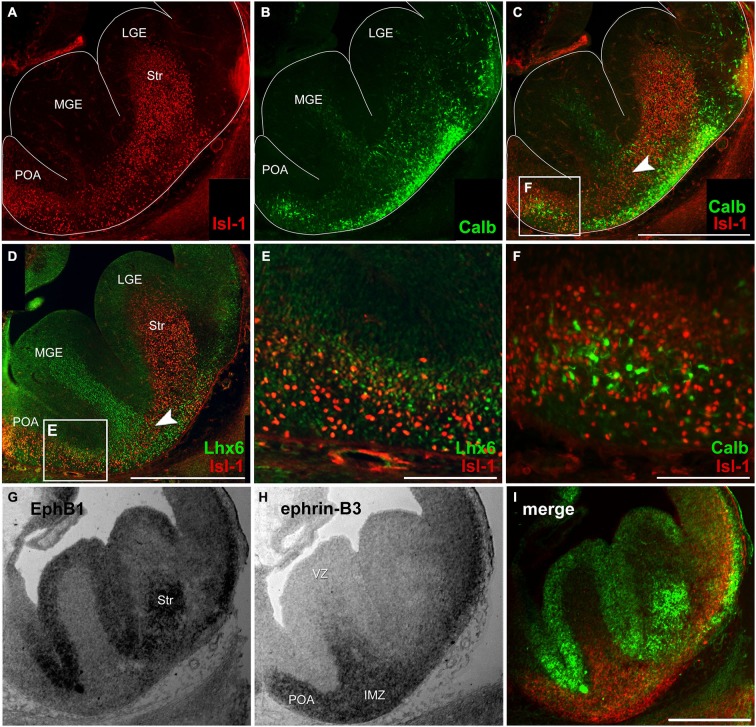
**Migratory pattern of cortical and striatal neurons and complementary mRNA expression of EphB1 and ephrin-B3 in the basal telencephalon.** **(A–C)** Double immunostaining against Isl-1 **(A)** and calbindin **(B)** on an E14 coronal hemisphere. Overlay of Isl-1 and calbindin immunostaining **(C)** reveals that there is no co-expression of both proteins **(F)** and illustrates the migration pattern of Isl-1 expressing striatal cells in the basal telencephalon as a narrow band in the transition zone (**C**, arrowhead) between the deep migratory stream (DMS) of cortical interneurons in the SVZ and the superficial stream in the IMZ, indicated by lower calbindin density. **(D–E)** Double immunostaining of Isl-1 and Lhx6 on an E14 hemisphere **(D)** shows no co-expression, too **(E)**. **(G–I)** *In situ* hybridization with EphB1 and ephrin-B3 riboprobes was performed on alternating coronal E14 brain slices reveals EphB1 labeling **(G)** of the developing striatum (Str) as well as the ventricular zone (VZ) of the lateral (LGE) and medial ganglionic eminence (MGE)—regions that are avoided by cortical interneurons. **(H)** Ephrin-B3 is strongly expressed in the POA and the intermediate zone (IMZ) ventral the striatum. **(I)** Pseudocolor overlay of **G** (green) and **H** (red) directly illustrates the complementary expression of EphB1 and ephrin-B3. Lateral is right and medial is left. IMZ, intermediate zone; LGE, lateral ganglionic eminence; MGE, medial ganglionic eminence; POA, preoptic area; Str, Striatum; VZ, ventricular zone. Scale bars: **(C,D,I)** 500 μm; **(E,F)** 100 μm.

**Figure 2:**
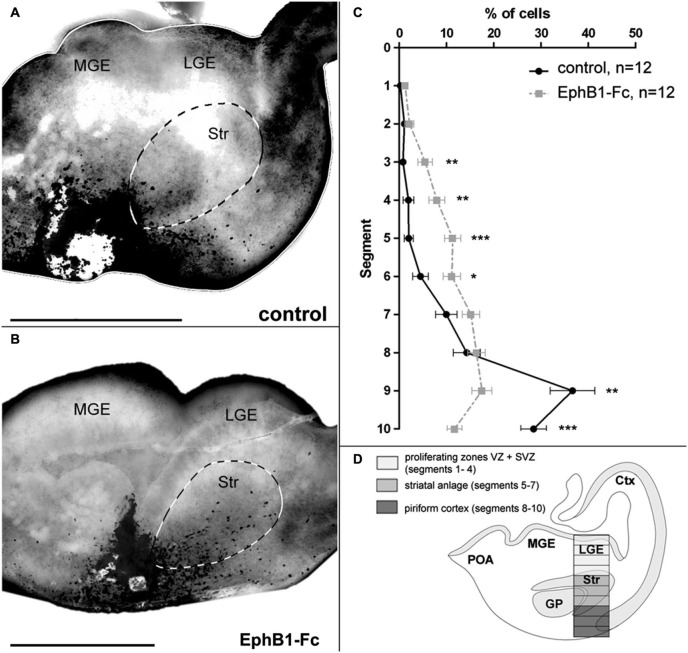
**Invasion of the striatum after blocking of endogenous ephrin-Bs in organotypic slice cultures**. The tangential migration of CellTracker C2925-labeled neurons of the superficial migratory stream in organotypic slices was investigated after blocking ephrin-B function. (**A**) Labeled SMS-cells in E14 slices after 1 DIV migrate mainly in the piriform cortex, avoiding the striatum in control conditions. (**B**) After blocking endogenous ephrin-B ligands with EphB1-Fc, labeled cells of the SMS invade the striatal anlage. (**C**) Quantitative analysis of the distribution of CellTracker C2925 labeled neurons at E14 plus 1 DIV under control conditions and after application of 5 μg/ml EphB1-Fc. (**D**) Schematic view of a coronal brain slice as used for the grafting experiments, illustrating the analyzed area which was horizontally divided into 10 equal segments, with segment 1–4 representing the proliferating zones, segments 5–7 the striatum and segments 8–10 the piriform cortex. Lateral is right and medial is left. GP, Globus pallidus; LGE, lateral ganglionic eminence; MGE, medial ganglionic eminence; SMS, superficial migratory stream; Str, Striatum. *n* = number of analyzed slices. Error bars indicate SEM. One-way ANOVA *p < 0.05, **p < 0.01, ***p < 0.001. Scale bars: 500 μm.

The distribution of the neurons on the stripes was determined using the cell counter plug in of *ImageJ*, whereas only the location of the soma was taken into account. Total numbers of neurons on the alternating stripes were corrected according to the varying widths of the stripes, and a paired* t*-test was used for statistical comparison. Results (mean ± SEM) are presented as a percentage; “n” refers to the number of analyzed pictures.

For the stripe assay combined with siRNA transfection, phase-contrast pictures were merged with photographs taken with fluorescence excitation, using the *Spot*-software, to visualize the transfected and non-transfected neurons per frame. The distribution of the neurons on the stripes was determined separately for transfected and non-transfected interneurons. A paired* t*-test was used for statistical comparison, results (mean ± SEM) are presented as a percentage; “n” refers to the number of analyzed pictures.

For quantification of the level of phosphorylated Src on dissociated Isl-1^+^ and Isl-1^−^ cells in the stripe assay images were taken using a confocal LSM. For each acquired image the fluorescence intensities of the pSrc signal of Isl-1 positive and negative cells growing on EphB1-Fc or control lines were measured using *ImageJ*. Signal intensities were then calculated relative to each other. Student’s *t*-test was used for statistical comparison. Results (mean ± SEM) are presented as a percentage; “n” refers to the number of analyzed pictures.

For the outgrowth assay the migration index was calculated by the area of outgrown cells relative to the area of the explants using *ImageJ*. Further the number of migrating Isl-1^+^ cells that left the explant, their migration distance from the edge of the explant as well as the migration distance of the three furthest migrated Isl-1^+^ and Isl-1^−^ cells was determined. The results of at least three independent experiments were analyzed (mean ± SEM); student’s *t*-test was used for statistical comparison, “n” refers to the number of explants.

### Quantitative analysis of the migratory streams

For quantification of the number of calbindin- and Lhx6-positive neurons in the Str the number of labeled cells was counted relative to the area of the Str that could clearly be identified with corresponding DAPI staining. Student’s *t*-test was used for statistical comparison; the number of analyzed brain sections is indicated as “n”.

For quantification of the immuno-reactive area the main range of labeled cells starting from the sulcus between the POA and the MGE was measured using *ImageJ*. Student’s *t*-test was used for statistical comparison; the number of analyzed brain sections is indicated as “n”.

For quantification of the relative fluorescence intensities of Isl-1-positive cells in the MGE and LGE, in both regions a column from the ventricle to the ventral border of the brain slice was analyzed with *ImageJ* (Figure 7H). For this, all sections of one area were reduced to the same pixel height with Adobe Photoshop CS3 before. The highest absolute fluorescence value was defined as 100% and the lowest value to 0%. In relation to these values the remaining relative fluorescence intensities were determined. Student’s *t*-test was used for statistical comparison; the number of analyzed brain sections is indicated as “n”.

**Figure 3:**
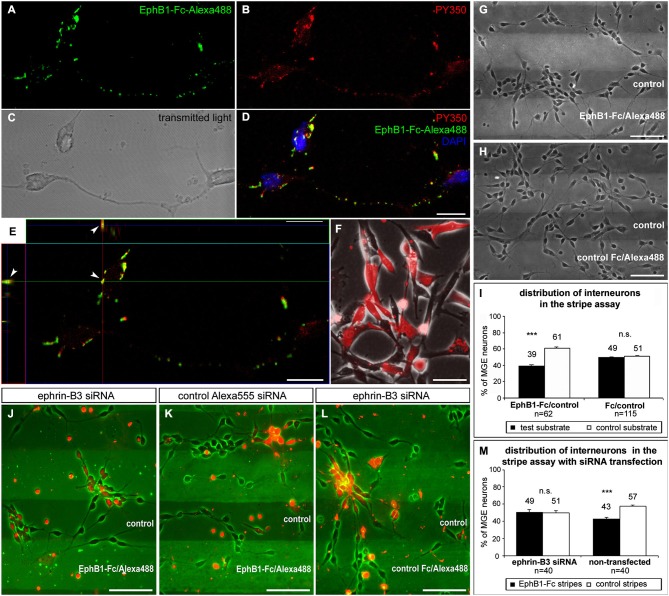
**EphB1 acts repulsive on neurons of the superficial migratory stream via reverse signaling.** **(A–D)** Stimulation of MGE neurons (**C**) with recombinant EphB1-Fc clustered with Alexa488 (**A,D**; green) leads to phosphorylation and thus activation of B-ligands as confirmed by immunostaining against phosphotyrosine PY350 (**B,D**; red). **(D)** Overlay of the PY350 immunostaining (red) with the EphB1-Fc binding sites (green) and nuclear staining with DAPI. (**E**) Co-localization of PY350 and EphB1-Fc-Alexa488 (arrowheads) as an evidence for effective ephrin-B-reverse signaling illustrated in an X and Y line scan through a single optical plane. (**F**) Ephrin-B3 expressing NIH3T3 fibroblasts co-transfected with ephrin-B3 siRNA and Alexa555-conjugated control siRNA. (**G**) Dissociated neurons from the MGE clearly avoid EphB1-containing lanes in the stripe assay after 2 DIV. (**H**) On alternating stripes of labeled and unlabeled control protein the cells show no preferential growth. (**I**) Quantification (mean ± SEM) of the distribution of MGE-derived neurons in the stripe assay with EphB1-Fc and control after 2 DIV. **(J)** After down regulation of ephrin-B3 ligands by siRNA transfection (red) in MGE-derived neurons the repulsion induced by EphB1-Fc in the stripe assay is abolished after 2 DIV, while non-transfected interneurons still avoid the EphB1-Fc stripes. (**K**) Application of Alexa555 labeled control siRNA has no effect as most of the cells still avoid the EphB1-Fc containing stripes. (**L**) Addition of ephrin-B3 siRNA does not affect MGE-derived neurons growing on Fc/control stripes. (**M**) Quantification (mean ± SEM) of the distribution of neurons in the stripe assay with and without ephrin-B3 siRNA transfection after 2 DIV. *Student’s t-test* ***p < 0.001. *n* = number of analyzed images. Scale bars: **(D,E)** 10 μm; **(F–H), (J–L)** 50 μm.

**Figure 4:**
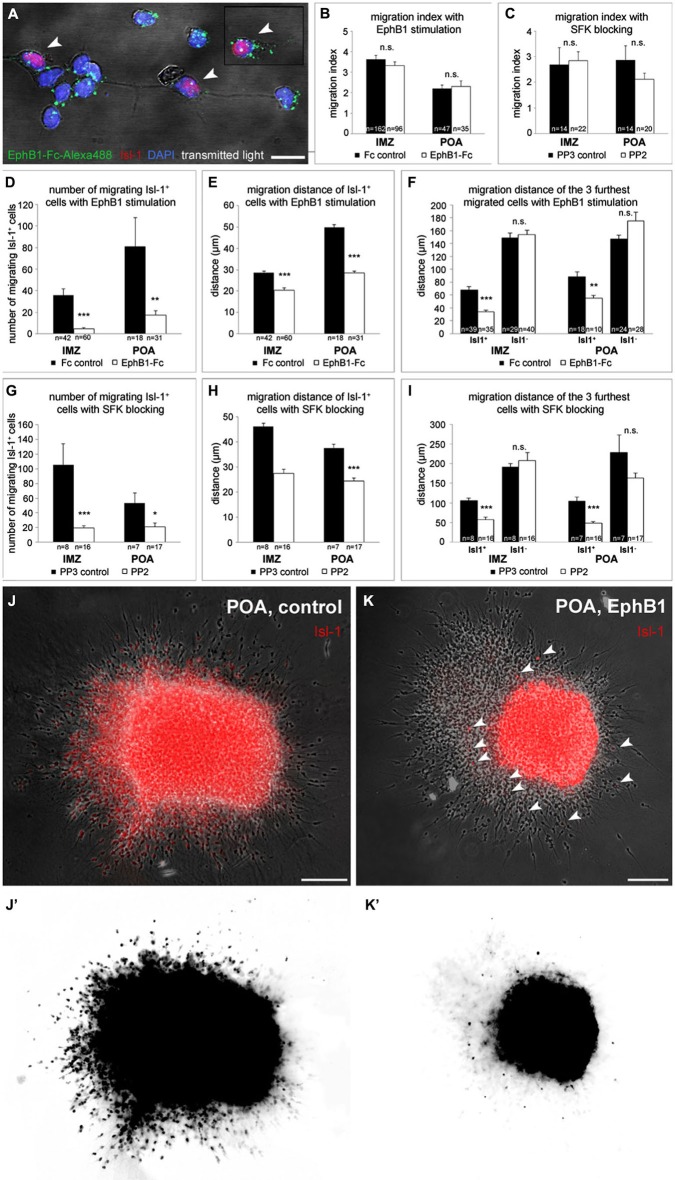
**EphB1 acts as a stop signal for Isl-1 expressing striatal neurons.** **(A)** Binding of recombinant EphB1-Fc clustered with Alexa488 to Isl-1 positive and negative dissociated neurons prepared from the IMZ. **(B–I)** Quantitative analysis (mean ± SEM) of the migration index (**B,C**), the number (**D,G**) and the migration distance (**E,H**) of migrating Isl-1^+^ neurons as well as the distance of the three furthest migrated cells (**F,I**) of IMZ- and POA-derived explants, respectively. Explants grew out in a three-dimensional substrate after application of EphB1-Fc or under control conditions (**B, D–F**) and with the Src inhibitor PP2 or the control protein PP3 (**C, G–I**), respectively. After treatment with EphB1-Fc or with PP2 less Isl-1^+^ cells leave the explant and if, they migrate not that far, while the general migration index of the explants and the migration distance of Isl-1^−^ neurons does not alter. **(J,K)** Overlay of phase contrast and Isl-1 immunostaining of POA-derived explants with (**K**) and without (**J**) EphB1-Fc treatment. **(J’,K’)** Photoconversion of the Isl-1 immunostaining of the explants shown in **J** and **K** illustrates the reduced number and distance of migrating striatal neurons (arrowheads) after application of EphB1-Fc (**K’**). Student’s *t*-test *p < 0.05; **p < 0.01; ***p < 0.001. *n* = number of analyzed explants. Scale bars: **(A)** 10 μm; **(J,K)** 100 μm.

**Figure 5:**
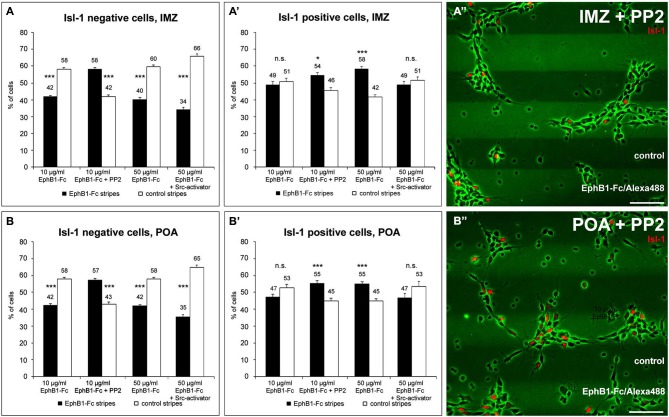
**The distribution of Isl-1 negative and Isl-1 positive neurons on EphB1 containing stripes is Src-dependent. (A–B’)** Quantification (mean ± SEM) of the distribution of Isl-1 negative and Isl-1 positive cells prepared from the IMZ (**A,A’**) and POA (**B,B’**), respectively, growing on alternating EphB1-Fc and control stripes containing 10 μg/ml EphB1-Fc (left, middle left) or 50 μg/ml EphB1-Fc (middle right, right). In some experiments 5 μM PP2 (middle left in **A–B’; A”–B”**) or 5 μM Src-activator (right in **A–B’**) were added. Student’s *t*-test *p < 0.05; **p < 0.01; ***p < 0.001. Scale bars: 50 μm.

**Figure 6:**
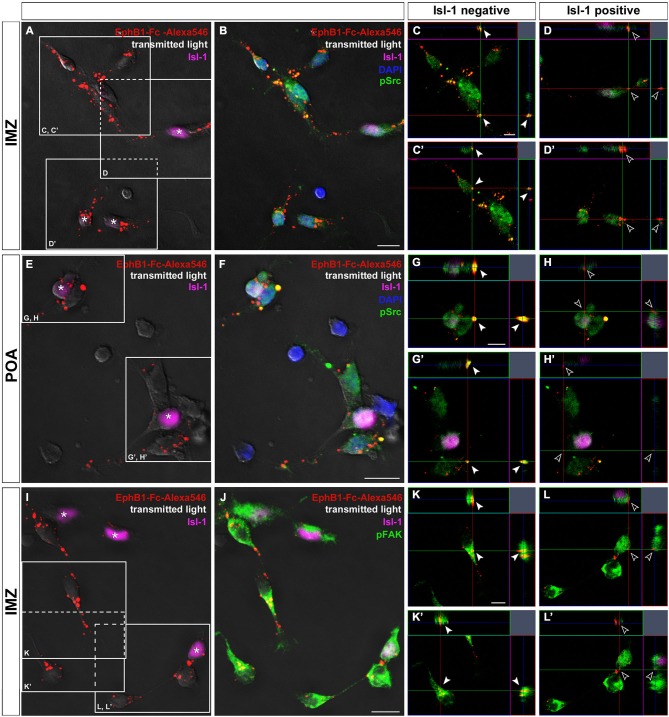
**Binding studies reveal differing activation of Src and FAK in neurons destined for the cortex or striatum**. Binding of Alexa546-labeled EphB1-Fc to neurons dissected of the IMZ (**A–D**) and POA (**E–H**), respectively. Single plane ortho-views (**C–D’** or **G–H’**) represent details of the main picture (**A–B** or **E–F**). Tripple immunostaining reveals co-localization of EphB1-Fc binding sites and pSrc signals, indicating activation of Src, only in Isl-1 negative neurons (arrowheads in **C,C’; G,G’**) while Isl-1 expressing cells (asterisks) show no or only weak Src activation as the merged image forms nearly no yellow signal (hollow arrowheads in **D,D’; H,H’**). The same result was found for co-localization of EphB1-Fc binding sites and pFAK signals, exemplarily shown for cells from the IMZ (**I–L’**). Scale bars: **(B,F,J)** 10 μm; **(C,G,K)** 5 μm.

**Figure 7:**
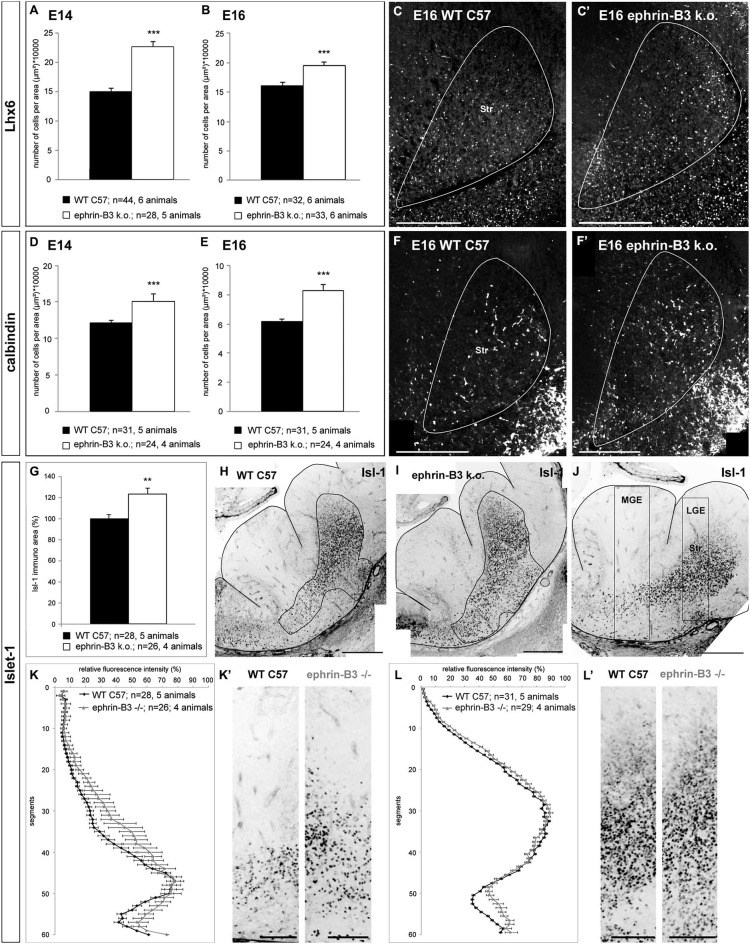
**The migration of cortical and striatal neurons in the basal telencephalon is affected in ephrin-B3 knockout mice. (A,B)** Quantitative analysis of the number of Lhx6 positive cells in the striatum at E14 (**A**) and E16 (**B**). **(C,C’)** Overlay of Lhx6 immunostaining and DAPI nuclear staining of the striatal region of a coronally sectioned hemisphere at E16. The white area marks the striatum. There are more Lhx6 stained neurons in the striatum of ephrin-B3 mutant embryos (**C’**) than in the wild type (**C**). The same result was found for calbindin positive neurons at E14 (**D**) and E16 (**E**). **(F, F’)** Overlay of calbindin and DAPI staining of a coronal slice at E16 at the region of the striatum in the wild type (**F**) and in the ephrin-B3 knock out (**F’**) indicating ectopic cells in the striatum. **(G)** Quantitative analysis of the area of stained Isl-1^+^ cells of E14 wildtype and ephrin-B3 knock out coronal slices as shown in **H** and **I**. The white region marks the measured immunoreactive area. **(J)** Isl-1 immunostaining of a coronal E14 WT slice in the orientation used for determination of the fluorescence intensities shown in **K–L’**. The white boxes represent the columns used for the plot profile in **K,K’** for the MGE and in **L,L’** for the LGE, containing the striatal anlage. **(K–L’)** Isl-1 expressing striatal neurons are misrouted in the ephrin-B3 knockout, indicated by increased mean relative fluorescence intensities around the transition zone of the MGE (**K,K’**) and in the piriform cortex superficial the striatum (**L,L’**). Student’s *t*-test ***p < 0.001. *n* = number of analyzed slices. Scale bars: **(C–F’)**, **(H–J)** 250 μm; **(K’,L’)** 100 μm.

## Results

### Migratory streams of cortical and striatal neurons in the basal telencephalon

At the peak of interneuron migration (E14 in the mouse), cortical interneurons born in distinct regions of the basal telencephalon follow separate routes when they migrate towards the cortex: an outer SMS and an inner or deep migratory stream (DMS; Corbin and Butt, 2011; Zimmer et al., 2011). These two migratory pathways are split by the striatal anlage, which is a non-target territory for cortical interneurons. On the other hand, the developing Str is the target of migrating striatal neurons, which are generated in the same regions and at the same developmental stages as cortical interneurons. In this study we want to decipher the cellular and molecular strategies that allow striatal neurons to enter and cortical interneurons to bypass the striatal anlage.

As a first step to characterize migrating striatal neurons we performed immunostainings of coronal brain sections at E14 using an antibody directed against the LIM-homeobox gene islet-1 (Isl-1), a marker for striatal precursor cells. During early development Isl-1 is expressed by both cholinergic and non-cholinergic striatal neurons while, as the differentiation progresses, Isl-1 expression is gradually restricted to cholinergic neurons and suppressed in non-cholinergic neurons (Wang and Liu, 2001). As shown in Figure 1A Isl-1^+^ neurons enter the Str from different directions: they are born in the LGE and MGE, but many neurons also originate from the POA. To visualize the routes of migrating cortical interneurons we performed immunostainings against Lhx6 and calbindin, both well-established early interneuron markers (Anderson et al., 1997; Lavdas et al., 1999; Polleux et al., 2002; Ang et al., 2003; Fogarty et al., 2007; Faux et al., 2010). Calbindin^+^ cells were found in both migratory streams: in the SVZ of the MGE and LGE which represents the deep route and, for neurons originating predominantly from the POA, in the IMZ, which represents the superficial migratory pathway (Figure 1B). Isl-1 and calbindin labeled cells that derive from the POA represent distinct populations of neurons since there is no co-expression of these two proteins (Figure 1F). Like calbindin, Lhx6 labeled neurons can also be found in both migratory pathways, with more Lhx6^+^ interneurons originating from the MGE than from the POA (Figure 1D). However, there is no co-expression of Isl-1 and Lhx6 (Figure 1E). Members of the Eph/ephrin family have been shown to act as cues that guide interneurons during their tangential migration (Zimmer et al., 2008; Rudolph et al., 2010; Zimmer et al., 2011). We performed *in situ* hybridization on coronal sections to examine the expression patterns of EphB1 and ephrin-B3 in the basal telencephalon at E14. EphB1 riboprobes revealed a strong signal in the Str and in the VZ of the ganglionic eminences and the POA. These are all regions that are avoided by migrating cortical interneurons. In contrast, at these developmental stages the ligand ephrin-B3 is expressed in the POA and in the IMZ, ventral of the Str, an area that is part of the SMS of cortical interneurons. The complementary expression patterns of EphB1 and ephrin-B3, illustrated by the pseudocolor overlay (Figure 1I), suggests that EphB1 might act as a repellent cue for cortical interneurons bearing the ephrin-B3 ligand. To test this hypothesis we performed the experiments described in the following sections.

### POA-derived neurons invade the striatum after blocking ephrin-B ligands

Previously we have shown that MGE- and POA-derived cortical interneurons migrate in spatially segregated corridors, in the deep and the superficial stream, respectively (Zimmer et al., 2011). Ephrin-B3, expressed in POA-derived interneurons traversing the superficial route, acts as a repellent signal for deeply migrating interneurons born in the MGE, which is mediated by EphA4 forward signaling. In contrast, EphA4 induces repulsive ephrin-B3 reverse signaling in interneurons generated in the POA, restricting this population to the superficial path. Since most cells in the superficial stream do not enter the Str, where EphB1 is expressed, and since most of these neurons bear the ephrin-B3 ligand, we designed an *in vitro* assay that allowed us to examine migrating cells of the superficial pathway whose ephrin-B ligands were blocked.

For this we performed experiments where tungsten beads coated with CellTracker C2925 were placed into the superficial stream in the IMZ at the edge of organotypic slices prepared from E14 mice to label migrating cells in this region. CellTracker reagents freely diffuse through the membranes of living cells where these probes react with intracellular components and produce a fluorescent product. After 1 DIV, the migration pattern of labeled cells in the basal telencephalon was examined. To interfere with the Eph/ephrin system, in some experiments we added EphB1-Fc to block endogenous ephrin-B ligands (Martínez and Soriano, 2005). As illustrated in Figure 2A, in control experiments the vast majority of tagged neurons in the superficial stream migrate through the IMZ and the piriform cortex towards the neocortex, thereby bypassing the Str. Only few cells invade this territory and are found in the Str after 1 DIV. In contrast, after blocking ephrin-B ligands by adding soluble EphB1-Fc to the medium, there is a clear increase in the number of labeled neurons entering the Str (Figure 2B). For a quantitative analysis we counted the number of marked neurons in defined segments of a column of the LGE (Figure 2D) as described in the methods section. As illustrated in Figure 2C, under control conditions most of the cells were found in segments 8 to 10 representing the piriform cortex (segment 9: **p < 0.01; segment 10: ***p < 0.001; one-way ANOVA; *n* = 12 slices), but not in the Str. However, in presence of soluble EphB1-Fc we detected significantly more labeled cells in segments delineating the striatal mantle zone compared to control experiments (segments 3–4: **p < 0.01; segment 5: ***p < 0.001; segment 6: *p < 0.05; one-way ANOVA; *n* = 12 slices). Thus, blocking membrane bound ephrin-B ligands at the surface of migrating interneurons leads to an invasion of these cells into the Str. In the experiments described in the next sections we characterize reverse EphB1/ephrin-B3 signaling in cortical neurons in more detail.

### Reverse EphB1 signaling repels many neurons of the superficial migratory stream

Using ephrin-B3 *in situ* hybridization combined with EphB1-Fc binding studies, we have previously demonstrated that about 80% of the calbindin-positive interneuron populations from the POA and SMS cells exhibit ephrin-B3 ligands (Zimmer et al., 2011). Here we combined EphB1-Fc binding with immunostaining against phosphotyrosine to demonstrate that back signaling from this receptor occurs via B-ligands expressed by interneurons. To determine this, we stimulated dissociated cells of the IMZ with EphB1-Fc coupled to Alexa488 to visualize EphB1 binding sites (Figure 3A; brightfield shown in Figure 3C). After immunostaining with antibodies directed against PY350 (Figure 3B), which demonstrate activation of target proteins at specific phosphorylation sites, we found phosphorylated tyrosines co-localized with Alexa488-labeled EphB1-Fc (Figure 3D). As illustrated in Figure 3E, an X-Y line scan through a single optical plane reveals co-localizations of EphB1-Fc binding sites and PY350. These data confirm that ephrin-B ligands became phosphorylated and thereby activated by EphB1, which is evidence for reverse signaling. Since EphB1-Fc preferentially binds to ephrin-B3 ligands in the SMS, we hypothesize that EphB1 expressed in the Str acts on ephrin-B3 bearing migrating cortical interneurons in a repulsive way to prevent them from entering this non-target territory.

To test this hypothesis, we used a stripe assay, where neurons dissected from the MGE were cultured on alternating stripes of labeled EphB1-Fc and unlabeled control protein. After 2 DIV, dissociated MGE neurons showed a preferential growth on the control stripes and the majority of the cells avoided the EphB1 stripes, as illustrated in Figure 3G. A quantitative analysis revealed that this effect was statistically significant (*p* < 0.001, paired *t*-test, Figure 3I). Thus EphB1 repels migrating neurons from the MGE via reverse signaling. In control experiments, with alternating stripes of labeled and unlabeled control protein, there was no preference of these cells for one kind of the stripes (Figures 3H,I).

### Down-regulation of ephrin-B3 abolishes the EphB1-induced repulsion of cortical interneurons

To confirm that ephrin-B3 mediates the repulsive effect of EphB1 on cortical interneurons, we transiently down-regulated ephrin-B3 ligands by siRNA transfection. For this, cultured MGE-derived cells in the EphB1-Fc stripe assay were transfected with siRNA directed against the mRNA of ephrin-B3 using lipofection. Additional application of fluorescence linked Alexa555 control siRNA allowed the visualization of the transfected interneurons. Thus, within the same stripe field, it was possible to directly compare the effect of EphB1 on transfected cells with repressed ephrin-B3 ligands and on non-transfected neurons with normal ephrin-B3 expression. The knockdown efficacy of the ephrin-B3 siRNA was verified in ephrin-B3 expressing NIH3T3 fibroblasts using RT-PCR. Normalized to the transfection rate and the actin expression level, we found a 53.1 ± 6.6% decrease (*n* = 3 independent experiments) in ephrin-B3 expression compared to control-transfected fibroblasts (Figure 3F).

As depicted in Figure 3J, down-regulation of ephrin-B3 ligands by siRNA transfection abolished the repulsive effect of EphB1, since transfected cells (labeled red) showed no preference for control stripes. In contrast, the majority of non-transfected cells with normal ephrin-B3 levels exposed to the same stripes avoided the EphB1-Fc containing lanes (Figures 3J,M). Application of the control Alexa555 siRNA alone showed no effect; in this case transfected as well as non-transfected cells avoided the EphB1-Fc stripes (Figure 3K). Moreover, application of ephrin-B3 siRNA had no impact on the distribution of the neurons on Fc/control stripes (Figure 3L). Thus, these results suggest that ephrin-B3 mediates the EphB1 response.

### EphB1 acts as a stop signal for neurons destined for the striatum

The experiments described above were performed using cells dissected of entire MGEs. Since striatal neurons also originate from the MGE and POA, in these experiments we obtained a mixture of cortical interneurons and cells destined for the striatum. To distinguish both cell types we used Isl-1 immunostaining to identify striatal neurons. We found that about 40% of all cells born in the POA express Isl-1. On their journey, POA-derived neurons split into separate streams, one destined for the striatum and one directed to the cortex. The cortical interneurons take the SMS and migrate in the most superficial aspect of the IMZ, while Isl-1 positive cells migrate deeper in the IMZ towards the striatum (Figures 1C,D, arrowhead).

As both cortical and striatal neurons are born in the same regions at the same developmental stages, we wondered how the striatum can be the target for one cell population and at the same time be a non-target region for the other population of cells. Obviously, there are several possibilities for how the striatum becomes a no-go area for cortical interneurons and a go area for striatal neurons. First, we hypothesized that cells destined for the striatum may simply not express B-ligands and therefore cannot respond to the repulsive EphB-1 signal expressed in the striatum. But performing an EphB1-Fc binding assay with cells obtained from the IMZ, we found that almost all Isl-1^+^ neurons also possess B-ligands (93.4%; Figure 4A). We then reasoned that striatal neurons might down-regulate their ephrin-B ligands just before entering the striatum, whereas cortical interneurons might bear ephrin-B3 all the time and therefore bypass the EphB1 expressing striatum. To address this question we performed EphB1-Fc binding assays with cells directly dissected from the striatum. By analyzing the number of Isl-1^+^ cells that bound Alexa488-linked EphB1-Fc, we found that nearly the same portion as in the SMS bound EphB1 (86.6%). Since ephrin-B1 shows only a weak expression in the POA and ephrin-B2 occurs mainly in the VZ of the LGE (Zimmer et al., 2011), these findings indicate that EphB1-binding sites on Isl-1 expressing striatal neurons refer predominantly to ephrin-B3. Thus, striatal neurons, even after entering their target, still express ephrin-B3 ligands.

We turned next to a cell migration assay to examine the effect of EphB1 stimulation on the two populations of neurons destined for the cortex and the striatum, respectively. Here, explants from either the POA or the IMZ were cultured in a clot of chicken plasma to allow cells to migrate in a three-dimensional substrate. After 20 h in culture, when the cells started to migrate out from the explants, in some experiments EphB1-Fc was then added to the medium. Then, the cultures were fixed after 2 DIV and an immunostaining against Isl-1 was performed to identify striatal cells. EphB1 stimulation did not affect the overall outgrowth of the explants, since the migration index of control and EphB1-Fc treated explants was identical. This was found with explants from both the IMZ and the POA (Figure 4B). But when we restricted our analysis to Isl-1^+^ cells, we discovered that only very few Isl-1 expressing cells migrate out of the explants and those that do so migrate over much shorter distances than Isl-1^−^ cells. For explants dissected from the IMZ, in control experiments, we counted a mean of 36 ± 6 Isl-1^+^ cells that left the tissue, while only 5 ± 1 Isl-1^+^ cells exited the explants after EphB1 application (Figure 4D). There were also several cases where only Isl-1 negative cortical interneurons, but no Isl-1 positive striatal cells migrated out of the tissue. Here Isl-1 staining could be detected only within the explants. Again we could see that EphB1 reduced the migration of Isl-1 positive cells: 93% of the explants showed outgrowth of Isl-1^+^ neurons under control conditions, whereas only 58% of the explants showed migration of Isl-1^+^ neurons after addition of EphB1-Fc. Moreover, the few cells that left the IMZ-explants in the presence of EphB1-Fc only migrated 20 ± 1.1 μm (*n* = 60 explants; 5 experiments), in contrast to 29 ± 0.7 μm under control conditions (*n* = 42 explants; 5 experiments; ***p < 0.001; student’s *t*-test; Figure 4E). The effect of EphB1 stimulation becomes even more pronounced in these experiments when one restricts the analysis to the three cells that migrated over the longest distances. Addition of EphB1 reduced the distance of the migrating Isl-1 positive cells by half, from 68 ± 4.4 μm under control conditions (*n* = 39 explants; 5 experiments) to 34 ± 2.4 μm (*n* = 35 explants; 5 experiments) with EphB1 stimulation (***p < 0.001; student’s *t*-test). In contrast, there was no difference in the migration distance of Isl-1^−^ cells in the presence or absence of EphB1 (149 ± 7.7 μm with Fc; *n* = 29 explants and 154 ± 6.8 μm with EphB1-Fc; *n* = 40 explants; Figure 4F).

For explants from the POA we obtained similar results. Under control conditions, in all cases examined, Isl-1^+^ were among the cells that migrated out from POA explants. After adding EphB1 to the medium, only 76% of the POA explants showed migrating Isl-1^+^ cells and less cells left the tissue (**p < 0.01; student’s *t*-test; Figures 4D,J,J’,K,K’) which reached only 28 ± 1 μm (*n* = 31 explants; 3 experiments) compared to 50 ± 1.1 μm in the control situation (*n* = 18 explants; 3 experiments; ***p < 0.001; student’s *t*-test; Figures 4E,J,J’,K,K’). We obtained similar results by comparing the three cells that migrated furthest from the POA explants. Again there was no difference between experimental and control conditions for Isl-1 negative cortical interneurons (147 ± 6.1 μm with Fc; *n* = 10 explants and 175 ± 13.6 μm with EphB1-Fc; *n* = 28 explants; Figure 4F).

Taken together, the experiments described so far for Isl-1^+^ cells destined for the striatum suggest that EphB1 acts as a stop signal for this set of neurons that keeps them in their target area. On the other hand, while EphB1 acts as a repulsive signal for Isl-1 negative cortical interneurons, it has no effect on the motility of these cells.

### EphB1 stops striatal neurons by reducing their endogenous pSrc level

To further examine the stop effect of EphB1 on striatal neurons, we reexamined the EphB1-Fc stripe assay, this time in combination with Isl-1 immunostaining. For this we dissected only cells of the IMZ and the POA that are part of the SMS. As expected from the previous stripe experiments, after 2 DIV Isl-1^−^ cortical interneurons of both regions examined avoided the EphB1-Fc containing stripes (Figures 5A,B; left). In contrast, Isl-1 positive neurons were equally distributed on the two types of stripes (Figures 5A’,B’; left). However, if EphB1 acts as a stop signal for Isl-1 positive striatal neurons, one would expect that Isl-1^+^ cells should be preferentially located on the EphB1-Fc stripes.

In attempt to resolve these discrepancies we examined EphB1 induced reverse signaling pathways in Isl-1^+^ and Isl-1^−^ cells. We first examined Src activation and used the Src inhibitor PP2 (Hanke et al., 1996) in the EphB1-Fc stripe assay. After blocking of Src the response to EphB1 of Isl-1^−^ cells from the IMZ and POA switched from repulsion, which is observed under control conditions, to attraction. As illustrated in Figures 5A,B (middle left) and Figures 5A”,B”, when PP2 was added, the majority of the neurons grew on the EphB1-Fc stripes: approximately the same ratio was *on* EphB1-Fc stripes as was *off* EphB1-Fc stripes without this inhibitor. Surprisingly, analyzing the Isl-1^+^ striatal cells that seemed not to respond to the EphB1-Fc stripes in the control situation, after blocking Src they also showed the same preference to EphB1 as did the Isl-1^−^ cortical interneurons (Figures 5A’,B’, middle left). Addition of the control peptide PP3 had no effect; the cells respond to the EphB1-Fc stripes as in the control experiments, without any treatment (data not shown).

Next we tested the effects of the FAK which in many cases is associated with Src. For this we used a pY397 antibody, since FAK becomes often autophosphorylated at Tyr397 when Src/FAK complexes are being formed. As illustrated in Figures 6J,K,L, confocal imaging revealed a co-localization of FAK (pY397) and Alexa488-labeled EphB1-Fc binding sites, indicating that binding of EphB1 also activates FAK in neurons of the IMZ. Both pSrc and pFAK immunostaining result in similar staining patterns and both show co-localization with EphB1-binding sites, suggesting that FAK and Src form a signaling complex. Due to the lack of suitable antibodies co-localization of pFAK and pSrc could not be directly examined. However, FAK blocking experiments showed the same results as the use of the Src inhibitor. To prevent the FAK autophosphorylation at the putative activation site, Tyr 397, we used the FAK inhibitor 14. The blocking efficacy of 3 μM FAK inhibitor 14 was verified using Westernblot (21% decrease of pFAK level; *n* = 4 independent experiments). After application of FAK inhibitor 14, in the stripe assay Isl-1^−^ cortical interneurons showed the same switch to attraction as they did with Src inhibitor PP2 (54.4 ± 0.9% on EphB1-Fc lanes; *p* < 0.001, paired *t*-test). Isl-1^+^ neurons also preferentially grew on EphB1-Fc stripes as they did in the presence of PP2 (58.4 ± 2.1% on EphB1-Fc lanes; *p* < 0.001, paired *t*-test; data not shown).

We next analyzed the level of phosphorylated Src on isolated Isl-1 positive and negative cells growing on EphB1-Fc lanes. Here, a double immunostaining against Isl-1 and pSrc was performed and the fluorescence intensities of the pSrc signal of Isl-1 positive and negative cells growing on EphB1-Fc or control lines were measured using *ImageJ*. We found that Isl-1^+^ striatal cells from the POA generally had a higher pSrc level than Isl-1^−^ cortical interneurons on control substrate. When Isl-1^+^ striatal neurons grew on EphB1-Fc stripes, the pSrc amount was reduced by 38.3% compared to the control stripes (**p < 0.01; student’s *t*-test). In contrast, Isl-1^−^ cortical interneurons showed no reduction of their pSrc level when they grew on EphB1-Fc lanes, to the contrary, they showed a slight increase (data not shown). We obtained similar results with dissociated cells dissected from the IMZ. Taken together, this data demonstrates that Isl-1^+^ striatal neurons have an overall higher Src expression than Isl-1^−^ cortical interneurons. When striatal neurons are exposed to EphB1 their pSrc level declines. In contrast, in cortical interneurons binding of EphB1 leads to activation of Src. Consistent with this proposal, we found a strong co-localization of EphB1 binding sites (Figures 6A–J) and pSrc (Figures 6B,F,J) in Isl-1^−^ cells from the IMZ or POA (Figures 6C,C’,G,G’,K,K’; arrowheads). In contrast, for Isl-1^+^ striatal neurons there is no or only very rarely a co-localization of pSrc with EphB1 binding sites (Figures 6D,D’,H,H’,L,L’; hollow arrowheads), suggesting that EphB1 does not induce Src phosphorylation in these cells.

Even though Isl-1 expressing striatal neurons showed no preferential growth in the stripe assay, by measuring fluorescence intensities we nevertheless could detect a significant decline in their pSrc level on EphB1-Fc stripes. Thus, perhaps the concentration of EphB1 used in this assay was not sufficient to reduce the pSrc levels enough to stop the Isl-1^+^ cells on the EphB1-Fc lanes. Therefore we reexamined this set of cells in the stripe assay, but this time we used a concentration of the EphB1 solution that was five times higher for preparing the stripes. As illustrated in Figures 5A’,B’ (middle right), using these higher concentrations now the majority of the Isl-1^+^ cells was located on the EphB1-Fc containing stripes. In the same experiments, Isl-1^−^ cells still avoided the EphB1 stripes and preferentially grew on the control stripes (Figures 5A,B; middle right).

Taken together, these data suggest that binding of EphB1 leads to a reduction of endogenous pSrc levels on Isl-1 expressing striatal neurons, which cause the cells to terminate their migration. To directly test this hypothesis, we used the outgrowth assay again, but this time we added the Src inhibitor PP2 instead of recombinant EphB1. In accordance with our hypothesis, reduction of Src levels led to strong reduction in the migration of Isl-1^+^ neurons, while Isl-1^−^ cells were not affected, similar to what we observed with EphB1 stimulation. Application of the control protein PP3 had no effect on the migratory behavior of the cells. Thus down-regulation of endogenous Src stops the migration of striatal (Figures 4G,H) but not cortical interneurons (Figures 4C,I). This suggests that for Isl-1^+^ cells, EphB1 has the same effect as addition of PP2: it reduces the level of phosphorylated Src in Is-1^+^ cells and thereby halts their migration. In contrast, EphB1 acts as a pure repellent cue for Isl-1^−^ cortical interneurons and reduction of pSrc by PP2 treatment mediates a switch from repulsion to attraction in the stripe assay, while neither EphB1 nor PP2 influence the motility of these cells.

Finally, we also performed experiments with increased pSrc levels using a Src activator, a phosphopeptide sequence (EPQYEEIPIYL) that activates Src family members by binding to their SH2 domains (Lu et al., 2009). The stripe assays described above suggested that the preference for the EphB1 stripes of Isl-1^+^ cells is caused by a reduction of pSrc levels. However, in the presence of a Src activator, striatal neurons no longer stop on the EphB1 lanes and instead become uniformly distributed over the two types of stripes (Figures 5A’,B’, right), while Isl-1^−^ cortical neurons avoid the EphB1 lanes (Figures 5A,B, right).

### Ectopic cortical interneurons in the striatum of ephrin-B3 knock-out mice

The *in vitro* data presented so far indicate that one single guidance cue, EphB1, can affect distinct cell populations in different ways. For cells destined for the striatum, EphB1 serves as a stop signal and thereby keeps these neurons in their target region. In contrast, for cortical interneurons, it acts as a repulsive cue preventing them from entering the developing striatum, an inappropriate target for this class of neurons. Strikingly, in both cases EphB1 induces these differential effects via ephrin-B3 reverse signaling. Therefore, to directly assess the effects of EphB1 on cortical interneurons and striatal cells *in vivo*, we examined these two sets of neurons in an ephrin-B3 knock-out (efnB3^−/–^) line. First, we performed immunostaining against calbindin and Lhx6 at E14 and E16 to label MGE- and POA-derived immature interneurons and counted the number of labelled cells in the striatum. For this analysis, we included only sections along the anterior-posterior axis which contained the POA, MGE and LGE.

As described above, in wild type mice, calbindin- and Lhx6-expressing neurons are found in the MGE and along the DMS, as well as in the POA and the superficial route (Figures 1B,D). Although the overall pattern of migrating cells seemed basically intact in the efnB3^−/–^ mice, we detected an increased number of ectopic cells in the developing striatum. For the quantitative analysis we counted the number of labeled neurons in the striatum in sections from wild type and ephrin-B3 mutant mice. Our quantitative analysis revealed that at E14, the number of Lhx6 positive neurons in the striatum increased from 15 ± 1 cells/10000 μm^2^ in the wild type brains (*n* = 44 sections from 6 brains) to 23 ± 1 cells/10000 μm^2^ in efnB3^−/–^ brains (*n* = 28 sections from 5 animals; ***p < 0.001; Figure 7A). In ephrin-B3 mutant brains, at E16, we also found more Lhx6 labeled cells in the striatum than in control brains (Figure 7B; section at E16 shown in 7C,C’). A similar result was obtained for calbindin positive cells in the striatum: more calbindin expressing neurons were present in the striatum of efnB3^−/–^ mutants in E14 as well as in E16 brain sections (Figure 7D for E14, 7E for E16; section at E16 shown in 7F,F’). The increased number of cortical Lhx6 and calbindin expressing interneurons in the developing striatum in the ephrin-B3 knock-out mice indicates a reduced repulsion of bypassing cortical interneurons, presumably via EphB1, which is expressed in this region.

### Misrouted striatal neurons in ephrin-B3 deficient mice

As the migration of cortical interneurons was affected in ephrin-B3 knock-out mice we also examined the migration pattern of striatal neurons. For this we performed immunostaining against Isl-1 at E14 to label striatal neurons. As indicated in Figures 1A,C,D, most Isl-1^+^ cells originate from the POA and migrate towards the striatum using a well-defined path in the transition zone between the DMS of cortical interneurons in the SVZ and the superficial stream in the IMZ (arrowheads). For quantification, we first measured the area of labeled Isl-1^+^ cells starting from the sulcus between POA and MGE as displayed in Figures 7H,I. The comparison showed that in ephrin-B3 deficient mice Isl-1 stained cells are more scattered and thus distributed over a 24 ± 5.7% larger area (*n* = 26 sections from 4 brains) than in the WT animals (*n* = 28 sections from 5 brains; **p < 0.01; Figure 7G). Furthermore, we measured the relative fluorescence intensities from the VZ to the SMS of the MGE and the LGE as indicated by the black boxes in Figure 7J, setting the highest value to 100% and the lowest value to 0%. The normalized plots revealed increased fluorescence intensity around the transition zone of the MGE in the efnB3^−/–^ mutants, indicating a higher number and wider distribution of striatal neurons in their migration path (Figures 7K,K’). This result also fits with our previous findings showing that bidirectional ephrin-B3/EphA4 signaling mediates the segregation of MGE- and POA-derived interneurons in the deep and superficial stream (Zimmer et al., 2011). The data presented here suggest that ephrin-B3/EphA4 signaling could also keep Isl-1 expressing neurons in their migration path to the striatum. If ephrin-B3 is missing in the mutant brain, this well-defined path is blurred. Plots through the LGE containing the striatum also revealed an altered distribution of Isl-1^+^ striatal neurons as we obtained increased fluorescence intensity in the piriform cortex where the SMS passes, while the deep boundary of the striatum remained nearly identical, anatomically restricted by the external capsule (Figures 7L,L’).

Taken together, deletion of ephrin-B3 not only alters the migration pattern of Isl-1^−^ cortical interneurons, as more cells invade the striatum than in the WT. It also has an impact on Isl-1 expressing striatal neurons, as they migrate in a more scattered way through the basal telencephalon and more cells were found outside the striatum compared to the WT animals.

## Discussion

The tangential migration of cortical interneurons from their origins in the basal telencephalon to the cortex has been the objective of numerous studies. Several families of brain wiring molecules, including semaphorins, slits, neuregulins and ephrins, contribute to regulating and orchestrating the precise translocation of these neurons from their birthplace to their distant target (Zhu et al., 1999; Marín et al., 2001; Wichterle et al., 2003; Flames et al., 2004; Nóbrega-Pereira et al., 2008; Rudolph et al., 2010; Zimmer et al., 2011; Rodger et al., 2012; Steinecke et al., 2014). Here we demonstrate that EphB1, which is expressed in the striatal anlage, repels POA-derived cortical interneurons by reverse signaling via ephrin-B3 and thereby prevents these neurons from migrating into an inappropriate region. In contrast, for striatal neurons, which are also generated in the MGE and the POA at the same developmental stages and also express ephrin-B3 on their surface, EphB1 has a different effect. For this set of neurons, EphB1/ephrin-B3 interaction leads to an arrest of cell migration.

Thus, these two populations of neurons with different destinations but with similar origins in location and in time and with similar receptor expression respond differently to the same guidance cue. We investigated the underlying mechanism of this apparent paradox and found that this dual action of EphB1 is due to differences in ephrin-B3 reverse signaling cascades in which the levels of phosphorylated Src and FAK are regulated differently. In Isl-1 expressing striatal cells, binding of EphB1 to ephrin-B3 leads to a reduction of the endogenously high pSrc and pFAK levels which causes the cells to terminate their migration. Accordingly, we found no co-localization of EphB1-binding sites with pSrc or pFAK in these cells. Pharmacological reduction of pSrc or pFAK levels mimicked the effects of EphB1 and thereby also led to a migration arrest. Conversely, in the presence of an increased pSrc level, the EphB1 effect was abolished and striatal neurons continued migration. In contrast, in cortical interneurons binding of EphB1 leads to phosphorylation of Src and FAK which mediates the repulsive effect of this guidance cue. Hence, we found co-localizations of EphB1-binding sites with pSrc as well as pFAK in these cells. After application of Src or FAK inhibitors, EphB1 response of cortical interneurons switched from repulsion to attraction. However, their general capability to migrate was not affected. Treatment with a Src activator had a synergistic effect with EphB1 on cortical interneurons, since EphB1 still appeared repulsive. Thus, for this set of neurons EphB1 acts as a guidance factor where activation or inhibition of Src determines about repulsion or attraction.

*In vivo* examinations in an ephrin-B3 knockout mouse line were consistent with these* in vitro* data. Due to the inadequate detection of the repulsive signal of the striatal anlage, homozygous ephrin-B3 knock-out mice showed an increase in misrouted Isl-1^−^ cortical interneurons in the striatum that were labeled by calbindin and Lhx-6, respectively. Furthermore, deletion of ephrin-B3 also has an impact on Isl-1^+^ striatal neurons, which show an over-migration due to the lack of their EphB1-mediated stop function that results in a more scattered migration pattern in the MGE and around the striatum.

### Multiple roles of the Eph/ephrin system during interneuron migration

Members of the Eph/ephrin family often act repulsively on migrating interneurons. For example, Zimmer et al. (2008) reported that calbindin expressing interneurons born in the MGE express EphA4 and are repelled by the ephrin-A5 expressing cells in the VZ of the ganglionic eminences that act as an inhibitory flank. Likewise, ephrin-A3 expressed in the striatum was shown to prevent the interneurons from entering this region (Rudolph et al., 2010). In both cases, repulsion was mediated by forward signaling via the EphA4 receptor and channeled MGE-derived neurons into the DMS. Ephrin-B3 expressing POA-derived interneurons also migrate within a spatially segregated corridor, traversing the superficial route. In this process repulsive bidirectional signaling mediated by EphA4 and ephrin-B3 leads to the segregation of these two streams (Zimmer et al., 2011).

Besides the conventional role of EphA4 to mediate repulsion, we recently also discovered that EphA4 has a cell type specific motogenic effect on migrating MGE-derived interneurons *in vitro* and *in vivo* (Steinecke et al., 2014). This effect was mediated by EphA4/ephrin-A2 reverse signaling, where EphA4 acts as a ligand on the migrating interneurons. Thus EphA4, which is known to guide MGE-derived interneurons to the cortex by forward signaling, there showed a different—now motogenic—effect on the same type of cells mediated by EphA4-induced reverse signaling.

In the present study we also saw distinct effects mediated by one receptor. Here EphB1 acts repulsive on cortical interneurons while it serves as a stop signal for striatal cells, both via ephrin-B3 mediated reverse signaling. Thus we found a novel function for a member of the Eph/ephrin system as a stop factor that was not described for this class before. In contrast to the migration promoting effect that EphA4 showed on cortical interneurons, we found a negative motogenic effect of EphB1 on migrating Isl-1 positive striatal cells.

### The impact of EphB1 on migrating neurons is mediated by FAK/Src signaling

Cells of both studied populations, Isl-1 positive and negative, bear ephrin-B3 on their surface but respond in different ways to the guidance cue EphB1, suggesting that these differences are downstream in the signal transduction cascade. We showed that phosphorylation of Src is necessary for the proper function of EphB1 as a stop or repulsive signal. When the constitutively high level of Src phosphorylated at position Tyr418 is reduced by dephosphorylation due to binding of EphB1, striatal cells arrest their migration. In contrast, in cortical interneurons activation or inhibition of Src causes repulsion or attraction, respectively.

One important interaction partner of Src is the FAK, which recruits Src into a FAK-Src signaling complex that enables the phosphorylation of various FAK-associated proteins. Due to its role in dynamic focal complex formation, FAK influences changes in actin and microtubule structures or affects cadherin-based cell-cell contacts and thereby promotes cell migration (reviewed in Mitra et al., 2005; Wu et al., 2008) demonstrated by the fact that FAK regulates Src via phosphorylation of position Tyr-418. This amino acid is located within the kinase domain and phosphorylation at this residue induces maximal Src activation.

Here we showed that the level of Src phosphorylated at this Tyr-418 is regulated differently in response to EphB1 in striatal and cortical cells which mediates the different effects of EphB1. The endogenous higher pSrc level in Isl-1 expressing striatal neurons suggested that FAK is more active in these cells. According to this hypothesis, the pFAK level in these neurons was enhanced compared to cortical interneurons (data not shown). After EphB1 stimulation we could not or only rarely identify a co-localization of pSrc or pFAK with EphB1 binding sites in Isl-1^+^ cells (Figure 6D). We therefore concluded that binding of EphB1 to its ligands leads to dephosphorylation of these FAK/Src-complexes in an unknown way and this decrease in activity terminates the migration of striatal neurons. It has already been shown that a reduction of FAK- or Src activity can have a negative influence on cell migration. This is in line with the stop effect described here. Due to deficient focal contact turnover, FAK-null fibroblasts show impaired migration and spreading, whereas they develop more stable focal adhesions (Ilic et al., 1995; Webb et al., 2004). Thus, FAK is necessary for the proper migration of cells and lack of FAK activity, like in FAK-null cells or by FAK dephosphorylation, hampers cell migration. Moreover, fibroblasts from mice deficient in the Src family members Src, Yes and Fyn, were found to display the same phenotype of reduced motility and spreading (Klinghoffer et al., 1999). This suggests that both, FAK and Src, are essential for the migration of striatal neurons, forming a FAK-Src signaling complex. If one of these or both proteins is deactivated by dephosphorylation via EphB1 binding or blocked by inhibitors as in the data presented here the migration of these cells is determined.

In contrast to striatal cells, binding of EphB1 in cortical interneurons led to phosphorylation and thus to activation of Src and FAK. This mediates the repulsive effect of EphB1 on this cell population. Blocking of Src or FAK function in this cell type converted the EphB1 response from repulsion to attraction but had no influence on the capability of the cells to migrate. This is reminiscent of the study by Zimmer et al. (2007) which showed that the response of cortical neurons to ephrin-A5 switches from repulsion to attraction after blocking SFKs with PP2.

Thus the different regulation of the endogenous pSrc- and pFAK-level determines the stopping of striatal cells or repulsion in cortical interneurons. The FAK/Src signaling cascade has already been shown to mediate a dual activity of a guidance cue: during positioning of the anterior commissure (AC), a major brain commissural projection, selective attractive and repulsive axonal responses to Sema3B mediated by FAK/Src signaling contribute to the formation of this axon tract. In this case it is not phosphorylation, but the integration of FAK/Src complexes into the membrane which determines the effect of this guidance factor. While phosphorylation of Src was detected in both types of axons, Sema3B induces a membrane recruitment of FAK/Src complexes only in anterior axons of the AC, which mediates an attractive response to Sema3B. In contrast, no such complexes were found in posterior axons of the AC on which Sema3B acts repulsively (Falk et al., 2005).

### Arresting migrating neurons

Applying several approaches we found that EphB1 expressed in the striatum arrests tangentially migrating striatal cells after they have reached their target area. This is a novel role for a member of the Eph/ephrin system since stop signals have thus far only been described for other systems. For example, the neurotransmitter GABA was found to stop tangentially migrating cortical interneurons after they entered their proper position in the cortex. Bortone and Polleux (2009) could demonstrate that ambient GABA, early in development, depolarizes migrating interneurons and promotes their ability to migrate. After they reached their cortical target area, the expression of the potassium-chloride co-transporter KCC2 is upregulated. This leads to a developmental switch from depolarization to hyperpolarization in response to GABA-binding, which prevents spontaneous intracellular calcium transients occurring in migrating interneurons. As a result, the pausing time of the neurons increases and migration finally stops. This mechanism requires a change of the properties of the migrating interneurons to halt the upregulation of KCC2. Since GABA is uniformly distributed in the developing cortex, the arrival of cortical interneurons in their target must be precisely coordinated with the expression of KCC2. How this is achieved is not known. In contrast, the results presented here indicate that Isl-1^+^ striatal cells bear ephrin-B3 constitutively during their migration. After they enter the striatum, they encounter EphB1. This acts as a stop signal for this population of neurons that terminates their migration and captures them in their target region.

Another example for a stop factor is the glycoprotein Reelin which halts radially migrating cortical neurons. Due to its role in cellular migration, Reelin plays a pivotal role in the correct formation of cerebral cortex layers (Chubb et al., 2008; Hernández-Miranda et al., 2010). In *reeler* mice lacking the Reelin protein, the orderly inside-out deposition of neocortical cells during development is disturbed resulting in inverted cortical layering (Caviness and Sidman, 1973). Reelin exerts its functions by binding to its receptors VLDLR (very low density lipoprotein receptor) and ApoER2 (apolipoprotein E receptor 2), which induces the phosphorylation of the adaptor protein Disabled-1 (Dab1) (D’Arcangelo et al., 1999; Howell et al., 1999) by Src family kinases (Arnaud et al., 2003; Bock and Herz, 2003). Phosphorylation of Dab1 is necessary for Reelin signaling as it is required for the detachment of neurons from radial glia which terminates neuronal migration (Sanada et al., 2004). Hence, inhibition of Dab1 phosphorylation by PP2-treatment was found to induce a *reeler* phenotype in cortical slice cultures (Jossin et al., 2003; Zhao and Frotscher, 2010). Recently, Sentürk et al. (2011) showed that ephrin-Bs, mainly ephrin-B3, are essential components of the Reelin pathway as a link between Reelin and Src to activate Dab1. Thereby Reelin not only binds to its receptors VLDLR and ApoER2 but also to the extracellular domain of ephrin-Bs. Activation of ephrin-Bs by EphB receptors then recruits and activates Src kinases which in turn phosphorylate Dab1 associated to the Reelin receptors at the membrane. Hence, loss of ephrin-B function showed an impairment of Reelin-mediated phosphorylation of Dab1. Conversely, Dab1 phosphorylation in *reeler* neurons could be recovered solely by activation of ephrin-Bs. Thus, neuronal migration defects normally resulting in *reeler* phenotype could be rescued in Reelin^−/–^ organotypic cortical slice cultures by activation of ephrin-B signaling (Sentürk et al., 2011). Thus, in cooperation with Reelin signaling, ephrin-Bs can also act as a stop signal during radial migration.

### Redundancy of guidance cues or cell type specificity?

The different parts of the basal telencephalon give rise to various discrete interneuron subtypes defined by a specific combinatorial code of intrinsic transcription factors (Butt et al., 2005; Flames et al., 2007; Miyoshi et al., 2007, 2010; Gelman et al., 2009). Thus, different subtypes of interneurons are already prespecified when they start to migrate and follow specific migratory pathways due to their internal program (Zimmer et al., 2011). Cortical interneurons born in the MGE migrate in the deep stream and mainly express EphA4 and neuropilin-1 (Nrp-1). In contrast, neurons originating from the POA mainly traverse the superficial migratory route and express ephrin-B3 and Nrp-2. The developing striatum represents a non-target territory for migrating cortical interneurons and expresses with class-3 semaphorins, as well as members of the Eph/ephrin system, multiple guidance cues that all act repulsively to cortical interneurons.

This might seem to be redundant as several cues performing the same function are expressed in the same region. From an evolutionary point of view, this redundancy might increase the reliability of the system as other guidance cues might compensate for developmental deficits. But cells in the same migratory stream belong to different subtypes that are specified by a combinatorial code of transcription factors that determine the configuration of downstream cell markers and guidance receptors that influence their migration. Due to this, different guidance systems of subpopulations of cortical interneurons are used to avoid entering the striatum. In this study we demonstrated repulsive ephrin-B3–EphB1 reverse signaling prevents cortical interneurons emanating from the POA to migrate into the striatum. In addition, it has been reported that cortical interneurons expressing Nrp-2 ligands that also migrate in the SMS bypass the striatum due to the repulsive effects of Sema3F (Marín et al., 2001). However, migrating MGE-derived interneurons in the DMS are repelled by Sema3A / Nrp-1 as well as by ephrin-A3 / EphA4 interactions (Marín et al., 2001; Rudolph et al., 2010). Thus different members of the Eph/ephrin and semaphorin family, the latter combined with CSPGs (Zimmer et al., 2010), all contribute to one function: keeping the striatum clean from cortical interneurons.

Furthermore, even in the same migratory stream cells that belong to different populations bearing different sets of guiding receptors respond to different guidance cues. For example, we found that only 15.7 +/− 1.3% of the cells in the superficial migratory route express Nrp-1 and only 22.7 +/− 0.9% Nrp-2, whereas almost all other cells in this region bear ephrin-B3 ligands (Zimmer et al., 2011). Finally, even if different subtypes of neurons express the same guidance receptors and are exposed to the same guidance signal, as in the case presented here, they can still respond differently to the same cue because different signaling cascades became activated in different sets of neurons. Considering that more than 20 different subtypes of cortical interneurons originate in the basal telencephalon (Xu et al., 2004; Wonders and Anderson, 2006) that all have to be guided on their way to the cortex, the multitude of guidance cues in the migratory pathways is probably more related to cell type specificity rather than redundancy.

## Conclusions

The results presented here, together with previous studies, demonstrate that members of the Eph/ephrin-system can act in various ways to guide different sets of migrating neurons through the basal telencephalon. Acting in forward and reverse, different Eph/ephrin combinations can delineate off-limits, preventing migrating neurons from invading inappropriate regions (Zimmer et al., 2008, 2011; Rudolph et al., 2010). Recently, we have also shown that EphA4/ephrin-A2 reverse signaling elicits motogenic activity on MGE-derived cortical interneurons (Steinecke et al., 2014). Here, we found that EphB1/ephrin-B3 reverse signaling acts as a stop signal for striatal neurons that keeps them in their target area. However, for cortical neurons, EphB1/ephrin-B3 reverse signaling leads to repulsion. Thus the same ligand / receptor combination can lead to very different physiological responses, depending on the downstream molecular machinery in the neurons.

## Conflict of interest statement

The authors declare that the research was conducted in the absence of any commercial or financial relationships that could be construed as a potential conflict of interest.

## Abbreviations

Acp: anterior commissure, posterior limb
BDNF: brain-derived neurotrophic factor
CGE: caudal ganglionic eminence
CSPG: chondroitin sulfate proteoglycan
Ctx: cortex
DAPI: 4′,6-Diamidino-2-phenylindol
DIV: days *in vitro*
DMEM: Dulbecco’s modified Eagle’s medium
DMSm: deep migratory stream
EGFP: enhanced green fluorescent protein
FAK: focal adhesion kinase
FBS: fetal bovine serum
GABA: γ-aminobutyric acid
GBSS: Gey’s balanced salt solution
GPI: glycosyl-phosphatidylinositol
HBSS: Hank’s balanced salt solution; Isl-1, Islet-1
LGE: lateral ganglionic eminence
MGE: medial ganglionic eminence
PBS: phosphate-buffered saline
PFA: paraformaldehyde
PP2: 4-Amino-5-(4-Chlorophenyl)-7-(t-Butyl)pyrazolo[3,4-d]Pyrimidin
PP3: 4-Amino-7-Phenylpyrazolo[3,4-d]Pyrimidin
SEM: standard error of the mean
Str: striatum
SFK: Src family kinase
siRNA: small interfering RNA
SMS: superficial migratory stream
SVZ: subventricular zone
VZ: ventricular zone.
